# Fibrillary glomerulonephritis: an observational study of clinical-pathological features and outcomes in patients from a multi-institutional cohort

**DOI:** 10.1093/ckj/sfaf166

**Published:** 2025-05-28

**Authors:** Jean Patrick, Madeline Charles-Rudwick, Charlotte Quinn, Anna Paterson, Lae T Soe, Ravi Varma, Oscar Swift, Gerald Glancey, Chintana Galahitiyawa, Lisa Wilcocks

**Affiliations:** Department of Renal Medicine, James Paget University Hospitals NHS Foundation Trust, Great Yarmouth, UK; Department of Renal Medicine, James Paget University Hospitals NHS Foundation Trust, Great Yarmouth, UK; Department of Medicine, Norfolk and Norwich University Hospitals NHS Foundation Trust, Norwich, UK; Department of Histopathology, Cambridge University Hospitals NHS Foundation Trust, Cambridge, UK; Department of Nephrology, Cambridge University Hospitals NHS Foundation Trust, Cambridge, UK; Department of Nephrology, Norfolk and Norwich University Hospitals NHS Foundation Trust, Norwich, UK; Department of Nephrology, East and North Hertfordshire NHS Trust, Stevenage, UK; Department of Nephrology, East Suffolk and North Essex NHS Foundation Trust, Ipswich, UK; Department of Nephrology, East Suffolk and North Essex NHS Foundations Trust, Colchester, UK; Department of Nephrology, Cambridge University Hospitals NHS Foundation Trust, Cambridge, UK

**Keywords:** DNAJB9, fibrillary glomerulonephritis, histopathology

## Abstract

**Background:**

Fibrillary glomerulonephritis (FGN) is a rare glomerulopathy characterized by randomly arranged fibrils within the mesangium and glomerular basement membrane. It has poor renal outcomes and no specific treatment. This retrospective study aimed to identify clinical-pathological predictors of outcomes in a multi-institutional cohort with histopathology performed at a single centre in the UK.

**Methods:**

Patients with biopsy proven FGN between 2015 and 2024 were identified using the Cambridge University Hospitals (CUH) histopathology database. Clinical data, including demographics, comorbidities, laboratory parameters, treatments and outcomes, were compared with findings from four other studies. Histological characteristics and DNAJB9 staining, where available, were analysed.

**Results:**

Thirty-five patients with FGN (2.8:1 female-to-male ratio) with a mean age of 62.8 years were identified. Autoimmune diseases, diabetes mellitus (DM) and malignancy (solid organ and haematological) were present in 28.5%, 34.2% and 20%, respectively. Nephrotic-range proteinuria was present in 58.8% and renal dysfunction in 91.4% at presentation. The most common histological pattern was mesangial proliferative/sclerosing followed by diffuse proliferative (DPGN). DNAJB9 staining was positive in all 13 tested patients. At a median 39 months follow-up, 51% progressed to end-stage renal disease (ESRD). Female sex, proteinuria and DPGN were significant predictors of ESRD on multivariate analysis. Although rituximab was associated with non-progression of disease, immunosuppression showed no statistically significant impact on outcomes.

**Conclusion:**

FGN is strongly associated with malignancy, autoimmune disease and DM. Prognosis remains poor, with progression to ESRD significantly influenced by proteinuria, DPGN and female sex.

## Graphical Abstract

**Graphical Abstract:**
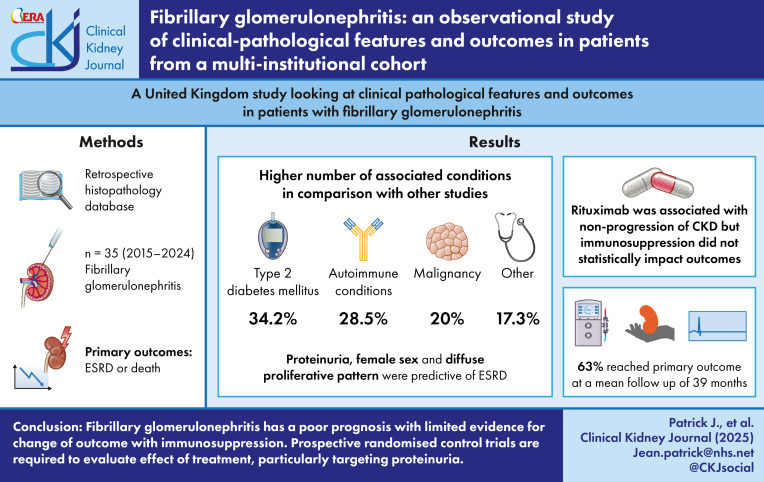

KEY LEARNING POINTS **What was known:**

- Fibrillary glomerulonephritis (FGN) is a rare and progressive kidney disease associated with poor outcomes.
- FGN has five histopathological patterns, and DNAJB9 is a biomarker sensitive and specific to FGN.
- FGN is characterized by significant proteinuria and progressive renal insufficiency, resistant to standard immunosuppressive therapies.

**This study adds:**

- Proteinuria, female sex and diffuse proliferative pattern on histopathology were identified as predictors of progression to end-stage renal disease.
- Rituximab may slow progression of disease, although in this study it did not show a statistically significant benefit.
- Subcategorization of mesangial sclerosis into proliferative and non-proliferative forms reveals distinct clinical characteristics.

**Potential impact:**

- Early targeting of proteinuria could potentially improve renal outcomes.
- These findings may shape future treatment protocols particularly involving rituximab.
- There findings may inform future treatment options for individuals with specific histopathological patterns and DNAJB9 positivity.

## INTRODUCTION

Fibrillary glomerulonephritis (FGN) is a rare, progressive form of glomerulonephritis first described in 1977 [1]. It is defined by the ultrastructural finding of randomly oriented straight fibrils measuring 10–30 nm in diameter by electron microscopy (EM) [2]. FGN is a histopathological diagnosis, and the incidence in native renal biopsies is <1% [3, 4]. Fibrils deposit in the mesangium and glomerular basement membrane. Immunofluorescence or immunohistochemistry stain intensely for immunoglobulin G (IgG) and complement 3 (C3), suggesting immune complex deposition. Five primary histological patterns have been identified: membranoproliferative (MPGN), mesangial sclerosing/proliferative (MES), membranous (MGN), diffuse proliferative (DPGN) and diffuse sclerosing (DS) [4, 5]. Crescents can be present in 17%–31% of cases [6]. The absence of staining for Congo red helps to differentiate FGN from amyloidosis in majority of cases. The diameter and the absence of a micro tubular appearance help differentiate FGN from immunotactoid glomerulopathy.

The discovery of DNAJB9, a protein involved in the stress response of endoplasmic reticulum, was a major breakthrough in understanding FGN pathogenesis [7]. DNAJB9 is found in the glomerular capillary wall and/or mesangium of patients with FGN. Its staining was found to have a sensitivity of 98% and specificity of 99% for the diagnosis of FGN [8, 9].

Patients with FGN typically present with nephrotic proteinuria, renal insufficiency and hypertension. Prognosis is poor, with approximately 50% of patients reaching end-stage renal disease (ESRD) within 5 years of diagnosis despite immunosuppressive therapy [4, 10].

To date, few studies have featured cohorts larger than 30 patients [4, 10–12]. In this study, we describe the clinical characteristics, histopathology, treatments, outcomes and associations with other conditions in a multi-institutional cohort from the East of England. We compare our findings with four large studies from the USA.

## MATERIALS AND METHODS

Patients with biopsy-proven FGN between 2015 and 2024 were identified from the Cambridge University Hospitals NHS Foundation Trust (CUH) histopathology database, which processes biopsies from seven regional hospitals throughout the East of England.

The diagnosis of FGN was defined as glomerular deposition of fibrils by EM examination with the following characteristics: randomly orientated non-branching fibrils 10–30 nm in diameter, a negative Congo red stain, and evidence of immunoglobulin and complement deposition. From 2020 onwards, DNAJB9 staining was also included in the criteria.

Data on demographics, clinical and laboratory findings, treatment and follow-up were extracted from electronic medical records. Nephrotic-range proteinuria was defined as >3.5 g/day. Quantification was performed by 24-h collection or by estimation using a spot urine albumin-to-creatinine ratio (uACR) or spot urine protein-to-creatinine ratio (uPCR). A uPCR of 100 mg/mmol or uACR of 70 mg/mmol was considered equal to approximately 1 g of proteinuria per 24 h. Interstitial fibrosis and tubular atrophy (IFTA) were graded on a semi-quantitative scale based on an estimate of the percentage of renal cortex affected and recorded as 0% (none), <25% (mild), 26 to 50% (moderate) or >50% (severe).

The combined primary outcome was ESRD or death. Secondary outcomes were modified from the Kidney Disease: Improving Global Outcome guidelines [13], and were classified as follows (i) complete remission (CR), defined as remission of proteinuria to <0.5 g/day (uACR <30 mg/mmol or uPCR <50 mg/mmol) with normal renal function; (ii) partial remission (PR), defined as reduction in proteinuria by >50% and to <2 g/day (uACR <140 mg/mmol or uPCR <200 mg/mmol) with stable renal function (≤20% increase in serum creatinine); (iii) persistent renal dysfunction (PRD), defined as failure to meet criteria for either CR or PR but not reaching ESRD, including patients with unremitting proteinuria or progressive chronic kidney disease; and (iv) ESRD, defined as an estimated glomerular filtration rate (eGFR) <15 mL/min/1.73 m^2^ or the need for renal replacement therapy or undergoing preemptive transplant.

All patient identifiable data was removed prior to analyses. As this study was a retrospective review of clinical data, formal approval from the National Health Service Research Ethics Committee was not required under national terms of guidance.

Statistical analysis was performed using Prism 10.0 (GraphPad) and SPSS V 30 (IBM Corp., USA). Parametric data were presented as mean ± standard deviation. Non-parametric data were presented as median (interquartile range). Comparison of two groups were performed with *t*-test or Mann–Whitney U analyses according to distribution. Categorical variables were analysed by Fisher's exact test or Chi-square test as appropriate and survival curves by Kaplan–Meier analysis. Cox proportional hazard regression was used to evaluate the association of potentially relevant covariates with the primary outcome. One-way analysis of variance (ANOVA) was used to compare means. A two-sided significance level of *P* < .05 was assumed.

## RESULTS

### Clinical characteristics

Thirty-five patients with a diagnosis of FGN were identified from a total of 4211 native renal biopsies (incidence 0.83%) by retrospective review of the histopathology database at CUH, from 2015 to 2024.

The cohort was 91% White, with a female predominance of 71% (Table 1). Mean age at biopsy was 63 ± 11.4 years. Renal insufficiency was present in 91.4% with a mean serum creatinine at presentation of 302 µmol/L. The mean level of proteinuria was 7.2 g/day and 58.8% had nephrotic-range proteinuria.

**Table 1:** Clinical characteristics of patients with FGN (*N* = 35).

Clinical parameters at time of biopsy	Result
Age, years, mean ± SD	63 (±11)
Female, *n* (%)	25 (71)
Race, *n* (%)	
White	31 (88)
African American	1 (3)
Asian	1 (3)
Unknown	1 (3)
Renal insufficiency, *n* (%)	32 (91.4)
Serum creatinine, µmol/L, mean ± SD	303 (± 372)
24-h proteinuria, g/day^a^, mean ± SD	7.2 (±11.2)
Proteinuria <1 g/day, *n* (%)	5 (14.8)
Proteinuria 1–3.5 g/day, *n* (%)	9 (26.4)
Proteinuria >3.5 g/day, *n* (%)	20 (58.8)
Serum albumin, g/L, mean ± SD	32 (±7.7)
Associated conditions, *n* (%)	
DM	12 (34.2)
Haematological malignancies^b^	2 (6)
Solid organ carcinomas^c^	5 (14)
Autoimmune disease	10 (28.5)
SLE	2 (6)
Rheumatoid arthritis	4 (11)
Graves’ disease	2 (6)
Sjögren's syndrome	1 (3)
Autoimmune thyroiditis	1 (3)
Hepatitis C	1 (3)

All results reported as mean (±SD) or number (%).

^a^Proteinuria data available for 34 patients.

^b^Non-Hodgkin's lymphoma (*n* = 1); lymphocytic leukaemia (*n* = 1).

^c^Lung cancer (*n* = 1); breast carcinoma (*n* = 1); colon carcinoma (*n* = 1); renal cell carcinoma (*n* = 1); melanoma (*n* = 1).

SD, standard deviation.

Autoimmune disorders were present in 28.5% of cases. The most common autoimmune disorder was rheumatoid arthritis (*n* = 4). Two patients had systemic lupus erythematosus (SLE) with no evidence of lupus nephritis histologically. Twelve patients (34.2%) had DM. There were five cases of non-haematological malignancies diagnosed over a period spanning 10 years before to 5 years after onset of renal disease. These included lung carcinoma (*n* = 1), colon carcinoma (*n* = 1), renal cell carcinoma (*n* = 1), and recurrence of breast carcinoma and melanoma (*n* = 1; same individual). Two patients had haematological malignancies. Antibody to Hepatitis C virus (HCV) was present in one patient. Demographic, laboratory and clinical characteristics were compared with four large studies from the USA (Table 2).

**Table 2:** Comparison of clinical characteristics of FGN patients in our cohort with those in other four large studies.

	This study (*n* = 35)	Nasr *et al.* [8] (*n* = 84)	Rosenstock *et al.* [4] (*n* = 61)	Schober *et al.* [11] (*n* = 42)	Andeen *et al.* [12] (*n* = 266)
Demographics					
Female (%)	71	74	61	60	65
Age, years	63 ± 11	59	56.8 ± 1.6	54 ± 10	61
Race (%)					
White	91	n/a	92	71	38.7
Black	3	n/a	5	26	3.3
Unknown	3	n/a	0	2	55.6
Asian	3	n/a	0	0	0.4
Hispanic	0	n/a	3	0	0.4
Comorbidities (%)					
Autoimmune disease	28.5	14	4.6	13	9
DM	34.2	24	20	28	22
Malignancy^a^	20	10	7	15	7.5
Hepatitis C	3	7	17	27	16
Dysproteinaemia	3	4	15 (7/46)	42 (8/19)	8
Clinical characteristics					
Renal insufficiency (%)	91.4	71	69	n/a	n/a
Mean creatinine, mg/dL	3.4	2.5	3.1	3.3	2.1
Mean proteinuria, g/day	7.2	5.1	6.4	5.7	n/a
Nephrotic-range proteinuria (%)	58.8	65	n/a	n/a	35

^a^Includes solid organ and haematological malignancies.

n/a: Not applicable.

### Histopathology

**Figure 1:**
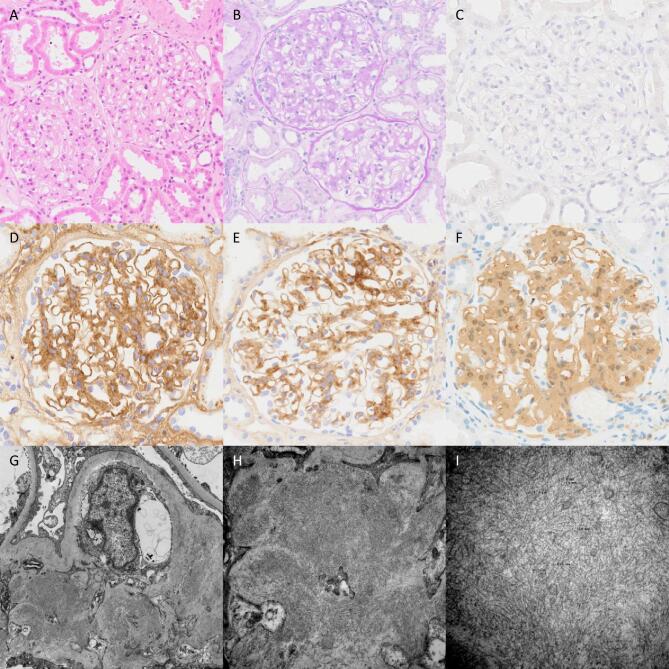
Two glomeruli showing a mesangial sclerosing/proliferative pattern of changes (**A**, H&E; **B**, PAS). A Congo red stain is negative (**C**). Granular mesangial and capillary wall staining is seen with IgG (**D**) and C3c (**E**), whilst a diffuse extracellular staining pattern is seen with DNAJB9 (**F**). Ultrastructural assessment shows the accumulation of randomly arranged fibrils in the mesangium and focally within the capillary wall with a diameter of 11.3 nm (**G**–**I**). H&E, haematoxylin and eosin; PAS, periodic acid–Schiff.

The average number of glomeruli examined on light microscopy was 20 (range 5–50). The most common histological pattern was MES, seen in 24 patients (68%) with varying degrees of interstitial inflammation and IFTA (Fig. 1). We were further able to classify the MES pattern as mesangial proliferative (*n* = 11) and mesangial sclerosis without proliferation (*n* = 13) subtypes to see whether there was any difference in outcomes in these subtypes. No statistical significant difference (*P* = .24) in outcomes were observed between these subtypes despite differences in mean creatinine, proteinuria and crescent percentage. One patient with MES pattern had antibodies against PR3 ANCA present in serum, however there was no crescent formation or fibrinoid necrosis present in the biopsy. The second most common pattern (17%) was DPGN (*n* = 6) in which there is mesangial and endocapillary hypercellularity with infiltrating mononuclear leukocytes (Table 3). Three cases (9%) exhibited an MPGN pattern.

**Table 3:** Histological findings and clinical correlations in FGN.

Histological pattern	No. of cases (*N* = 35) (%)	Mean creatinine (*n* = 35) (SD)	Mean albumin (*n* = 35) (SD)	Mean proteinuria g/day (*n* = 34) (SD)	% Crescents	Moderate/severe interstitial disease, *n*/*n* (%)
MES	13 (37)	240.5 (±181.75)	30.38 (±8.12)	9.18 (±16.93)	0.3	5/13 (38)
MESP	11 (31)	165.5 (±105.58)	37.27 (±6.68)	3.1 (±2.77)	3.30	3/11 (27)
MPGN	3 (9)	947.5 (±1205.62)	33 (±11.31)	14.18 (±11.76)	8.6	1/3 (33)
DPGN	6 (17)	580.3 (±455.42)	29.17 (±4.14)	10.2 (±4.56)	26.0	4/6 (66)
DS	1 (3)	84 (n/a)	41 (n/a)	2.63 (n/a)	0.0	1/1 (100)
MGN	0 (0)	n/a	n/a	n/a	n/a	n/a
Normal pattern	1 (3)	93 (n/a)	22 (n/a)	8.17 (n/a)	0.0	0/1 (0)
ANOVA		*P* = .113	*P* = .069	*P* = .776	*P* = .723	*P* = .487

MESP, mesangial sclerosis with proliferation; SD, standard deviation.

n/a: Not applicable.

There was one case with a DS pattern, which exhibited severe glomerular and arteriolar sclerosis. There were no cases of MGN and in one case the glomeruli showed no significant abnormalities on light microscopy, however the diagnosis of FGN was confirmed by identification of randomly arranged fibrils on EM and no Congo red staining.

DPGN pattern was associated with increased ESRD incidence (*P* = .002) and reduced time to ESRD (*P* = .035) compared with other patterns.

Cellular or fibrocellular crescent formation were present in 31% of cases. Crescents were most frequently seen in DPGN (five out of six cases) and when present, involved a median of 29% (5.3%–57%) of glomeruli. Crescents were infrequently observed in the MES and MPGN patterns and absent in DS pattern of injury.

The degree of IFTA ranged from absent (14%) to mild (40%) to moderate (23%) to severe (17%), and 6% of reports did not have this stated. The DS case had severe IFTA. All biopsies in the diffuse proliferative subgroup had some degree of IFTA with 66% showing moderate to severe fibrosis. Biopsies of patients in the MPGN and MES subgroups had 33% IFTA.

Immunohistochemistry was performed in 34 out of 35 cases and 85% of cases stained positive for IgG. IgG4 subclass stained positive in 76% cases, and this was followed by 55% positive for IgM and 35% for IgA. Staining for IgG1, IgG2 and IgG3 subclasses were not undertaken. C1q was detected in 82% of cases and C3 in 70% of cases. Thirteen out of the 35 cases (37%) were stained for DNAJB9 and all of them were positive. All cases exhibited negative Congo red staining.

EM (*n* = 34) showed randomly oriented and non-branching fibrils in all cases. The mean diameter of fibrils measured in 32 cases (91%) was 14.8 ± 5.2 nm.

### Treatment

Seventeen patients (48%) did not receive immunosuppression (IS). Of these, eight reached ESRD, seven had PRD and two patients achieved spontaneous CR at the time of last follow-up. Eighteen patients had received IS at the time of last follow-up. Most patients received multiple IS agents. Four received prednisolone monotherapy. Rituximab was administered to five patients, of whom two received concomitant steroids, and one patient had previously received cyclophosphamide, mycophenolate mofetil (MMF) and tacrolimus. Five received MMF and three patients received cyclophosphamide in combination with steroid therapy. In almost all cases IS was targeted to treat the FGN except in one patient who received adalimumab with methotrexate to treat rheumatoid arthritis.

The type of IS administered differed between histological subtypes. Steroid monotherapy (*n* = 4) and rituximab (*n* = 2) was given to patients with MES pattern but specifically to ones without proliferation. MMF (*n* = 3) and rituximab (*n* = 1) were offered to patients with MES with proliferation. Cyclophosphamide (*n* = 3) and MMF (*n* = 1) were offered to patients with DPGN pattern.

For any histological subgroup the use of IS did not correlate with outcomes such as incidence of ESRD or time to ESRD. IS did not slow the progression to ESRD by Kaplan–Meier survival estimates (*P* = .748). Of the four patients treated with steroids, three (75%) reached the primary outcome of ESRD at a median follow-up time of 23 months. Of the six patients who received MMF, three (50%) reached ESRD at a median follow-up of 40 months. All three patients who received cyclophosphamide reached the primary outcome of ESRD at a median follow-up of 70 months. Of the five patients who received rituximab only one reached ESRD at a median follow-up time of 26 months. The remaining four patients had PRD, but the observed benefit was not statistically significant (hazard ratio 0.26, confidence interval 0.03–2.14, *P* = .21). The median baseline serum creatinine and proteinuria were lower in the rituximab group compared with the other IS group but this difference did not reach statistical significance.

### Outcomes

**Figure 2:**
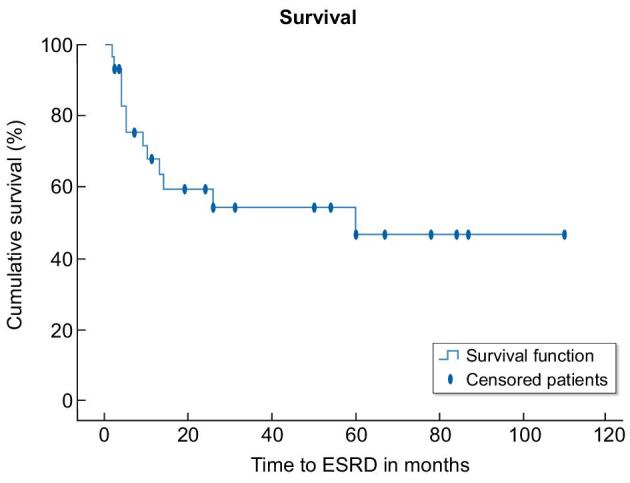
Kaplan–Meier survival analysis in FGN.

**Figure 3:**
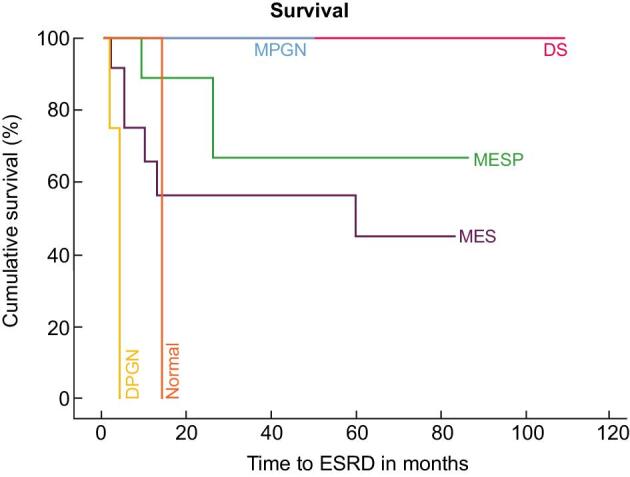
Kaplan–Meier survival analysis in histological subtypes of FGN.

At a median duration for follow-up of 39 months (12–70 months), 63% reached the primary outcome of ESRD or death. At the last follow-up, two patients (6%) had CR, three patients (9%) had PR, 12 patients (34%) had PRD and 18 patients (51%) had progressed to ESRD (Table 4). The median time to ESRD was 5 months (Fig. 2). Of the 18 patients who reached ESRD only 5 received a kidney transplant, and at the time of last follow-up no recurrence of disease in the graft was reported in these patients. Ten patients (29%) had died, of which seven had reached ESRD, two with PRD and one with PR. The mean time to ESRD varied according to histological subtypes, (Fig. 3) but did not show statistical significance (*P* = .097) in this small cohort. Excluding the five patients with ESRD at presentation, on univariate analysis by Cox regression, predictors of reaching ESRD (Table 5) were serum creatinine (*P* = .014), eGFR (*P* = .034), proteinuria (*P* = .005) and DPGN (*P* = .002). All six cases of DPGN reached ESRD. On multivariate analyses, proteinuria (*P* = .05) female sex (*P* = .021) and DPGN pattern (*P* = .014) remained significant predictors of ESRD (Table 6).

**Table 4:** Treatments and outcomes of patients with FGN in our study compared with those in other cohorts.

	This study (*n* = 35)	Rosenstock *et al.* [4] (*n* = 61)	Nasr *et al.* [10] (*n* = 61)	Schober *et al.* [11] (*n* = 42)	Andeen *et al.* [12] (*n* = 100)
No IS, *n* (%)	17 (49)		16 (26)	3 (7)	70(70)
Immediate haemodialysis, *n* (%)	5 (14)			3 (7)	6 (6)
RAS blockade alone, *n* (%)	10 (29)		16 (26)	10 (24)	
IS, *n* (%)	*n* = 18 (51)	*n* = 20 (33)	*n* = 29 (44)	*n* = 20 (48)	*n* = 30 (30)
Steroids alone	4 (22)	9 (45)	8 (28)	1 (5)	
MMF	5 (28)		6 (21)	1 (5)	6 (20)
Rituximab	5 (28)		3 (10)	15 (75)	8 (23)
Cyclosporine		3 (15)	2 (7)	4 (20)	
Cyclophosphamide	3 (16)	8 (40)	9 (31)	2 (10)	9 (30)
Steroids included	9 (50)		24 (83)	8 (40)	
Tacrolimus	2 (5)				
Outcome, *n* (%)					
Dialysis/ESRD	18 (51)	28 (45)	27 (44)	31%	42 (42)
Transplant	5 (14)	2 (3)	14 (23)	10%	11 (11)
Death	10 (27)		12 (20)	24%	13 (13)
Complete remission	3 (9)		3 (5)		1 (1)
Partial remission	2 (6)		5 (8)		18 (18)
Persistent renal dysfunction	12 (34)		26 (43)		18 (18)

RAS, renin–angiotensin system.

**Table 5:** Univariate analysis for ESRD correlates.

Variable	*P*-value
Female sex	.1
Age at diagnosis	.35
Auto immune conditions	.37
Serum creatinine	.01
Proteinuria	.005
Serum albumin	.3
Fibrosis	.39
Crescents	.88
eGFR	.03
DPGN	.002

**Table 6:** Predictors of reaching ESRD on multivariate analysis.

Variable	Hazard ratio	95% confidence interval	*P*-value
Female sex	11.23	1.43–88.14	.021
Proteinuria	1.13	0.99–1.28	.052
DPGN	1049.43	4.13–266 261.68	.014

## DISCUSSION

In this multi-institutional UK cohort, we report the clinical features and outcomes of patients with FGN in the East of England between 2015 and 2024 and compared our findings with four other studies from the USA.

Our cohort showed a female predominance (71%) and a mean age of 63 years at diagnosis, which is older than the weighted average of 57.7 years reported in other studies [5, 10, 12]. The strong White racial predominance reflects the ethnic makeup of the region. Association with autoimmune disease, DM, hepatitis C and malignancies are reported with FGN [5, 10]. In our study, higher rates of autoimmune disorders (28.5%) and DM (34.2%) were noted compared with previous studies. There is no published data suggesting a higher prevalence of autoimmune disease or DM in our region so this could merely be a chance variation given the small sample size.

The mean proteinuria at the time of biopsy was 7.2 g/day and 91.4% of patients had renal insufficiency with a mean creatinine of 302 µmol/L. These values are higher than reported in other studies indicating that our patients were diagnosed later in the course of their disease.

Amongst the five different histological patterns observed in FGN, with the exception of one study in which MPGN was the predominant histopathological variant, MES has been the most common pattern reported so far [3, 4, 6, 8]. This is characterized by solely mesangial proliferation and expansion in the absence of involvement of the glomerular capillary lumen. MES was also the most common (68%) pattern seen in our study. We were further able to histologically subclassify the MES pattern into mesangial sclerosis without proliferation and those with proliferation. Despite not seeing a significant difference in outcomes between the MES subtypes, we recommend that MES be classified based on the presence of proliferation which may help guide future treatment protocols.

In our cohort, crescents were present predominantly in the DPGN pattern. While DPGN was significantly associated with ESRD, presence of crescents themselves did not reach statistical significance as an independent predictor. This may be due to their distribution across other histopathological patterns with non-progressive disease.

In recent years, DNAJB9 has emerged as a biomarker for FGN, with high sensitivity and specificity [8, 11, 14, 15]. In our cohort, all 13 patients who were diagnosed since 2020 had positive staining for DNAJB9. This biomarker has advanced our understanding and diagnostic accuracy in FGN.

Predictors of ESRD progression have varied across studies. Rosenstock *et al.* identified serum creatinine and interstitial disease as significant predictors of ESRD in multivariate analysis [4]. The Mayo clinic study highlighted age, proteinuria, creatinine and % of glomerulosclerosis as key factors [10]. Interestingly, a study by Andeen *et al.* found male sex and an eGFR <45 mL/min/1.73 m^2^ to be predictors, with males showing a non-significant trend towards HCV and higher eGFR [12]. In our cohort, female sex did not initially emerge as a significant predictor, it became significant in the multivariate model, suggesting an independent association with the outcome.

In this small retrospective study, there was no statistically significant improvement in outcome for the 51% patients who received IS. Since FGN involves the glomerular deposition of immunoglobulin, there is a rationale for the use of the CD20-positive B-cell depleting agent rituximab. Although published outcomes of treatment with rituximab vary, they have shown stabilization and non-progression to ESRD in patients treated with rituximab [16–18]. Similarly, in our cohort, rituximab was associated with non-progression to ESRD in four out of five patients. However, the lack of statistical significance is likely due to the small sample size in this very rare disease. One patient with FGN and a history of SLE achieved partial remission after switching from azathioprine to MMF [19].

We acknowledge the limitations in this study are its observational and retrospective nature, incomplete Kappa/Lambda and DNAJB9 staining across all cases and heterogeneity in treatment regimens. All data were gathered from medical records and clinician discussions, and there were some incomplete data particularly related to follow-up. However, when compared with previously reported large studies, ours has comparable clinical pathological findings and outcomes.

In conclusion, the findings confirm the poor prognosis of FGN even with IS therapy. After a median follow-up of 39 months, 51% reached ESRD. In multivariate analysis female sex, proteinuria and DPGN emerged as significant predictors of ESRD. While rituximab showed a trend towards better renal outcomes, this was not statistically significant and warrant further investigation in larger cohorts.

## Data Availability

The data that support the findings of this study are available from the corresponding author upon request. By recommendation of the ethics committee, it is required that the purpose for which this information is to be used and those with whom it is to be shared are given at the time of the request as well as an undertaking that the confidentiality of the patients included in the study will be maintained.
